# Supplementary figures and images for: Signatures of Co-Deregulated Genes and Their Transcriptional Regulators in Lung Cancer
Source: Int J Mol Sci. 2022 Sep 18;23(18):10933. doi: 10.3390/ijms231810933 (PMC9504879; doi:10.3390/ijms231810933)

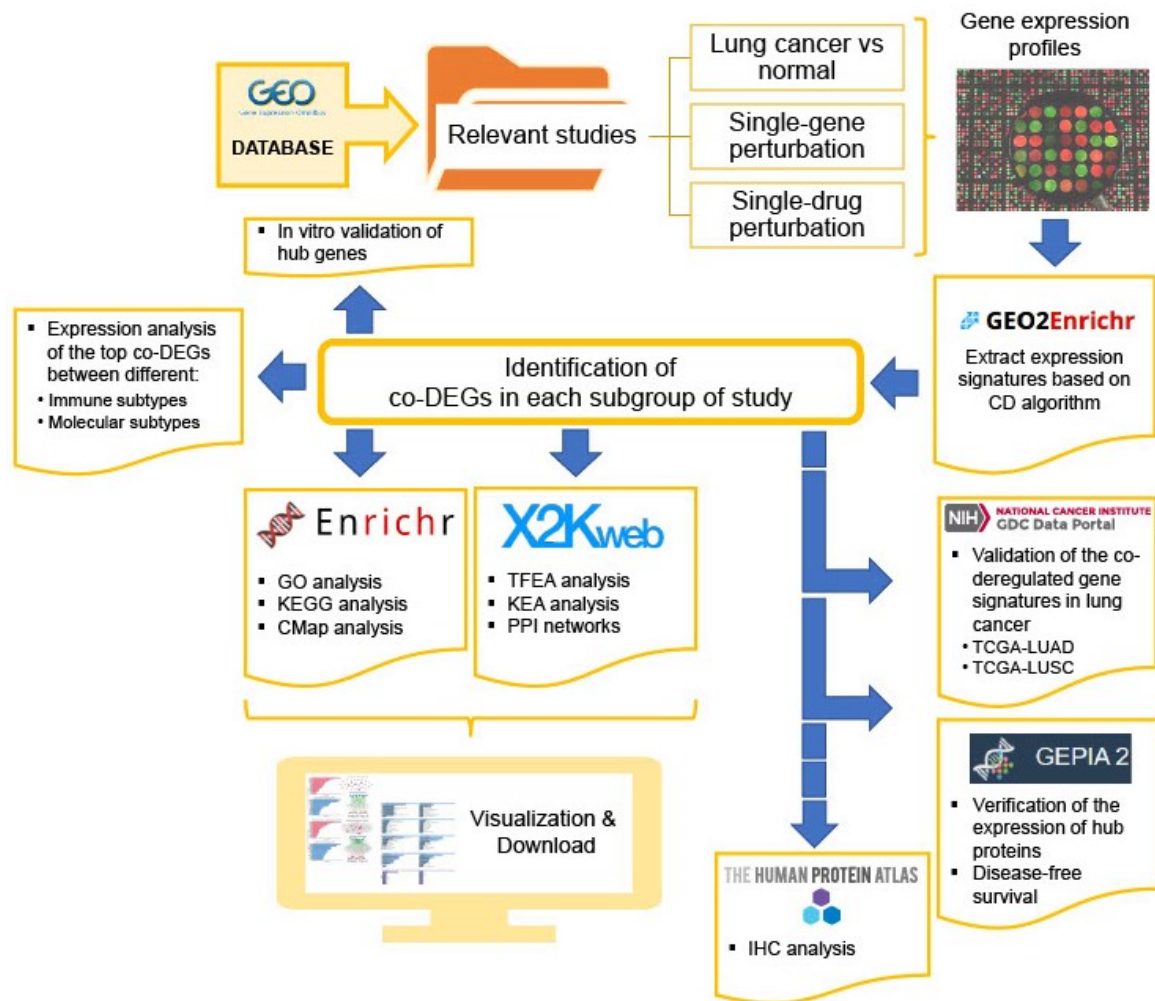

**Figure S1.** Flow chart explaining the analytical process followed.

Supplement: Supplementary file 1 [file ijms-23-10933-s001.zip › ijms-1930414-Figure S1.pdf]
